# The BROAD study: A randomised controlled trial using a whole food plant-based diet in the community for obesity, ischaemic heart disease or diabetes

**DOI:** 10.1038/nutd.2017.3

**Published:** 2017-03-20

**Authors:** N Wright, L Wilson, M Smith, B Duncan, P McHugh

**Affiliations:** 1 Royal New Zealand College of General Practitioners, Gisborne, New Zealand; 2 Two Zesty Bananas Limited, Wellington, New Zealand; 3 BROAD Study, Gisborne, New Zealand; 4 Hauora Tairāwhiti, Gisborne, New Zealand; 5 Department Primary Health Care and General Practice, University of Otago, Wellington, New Zealand

## Abstract

**Background/Objective:**

There is little randomised evidence using a whole food plant-based (WFPB) diet as intervention for elevated body mass index (BMI) or dyslipidaemia. We investigated the effectiveness of a community-based dietary programme. Primary end points: BMI and cholesterol at 6 months (subsequently extended).

**Subjects:**

Ages 35–70, from one general practice in Gisborne, New Zealand. Diagnosed with obesity or overweight and at least one of type 2 diabetes, ischaemic heart disease, hypertension or hypercholesterolaemia. Of 65 subjects randomised (control *n*=32, intervention *n*=33), 49 (75.4%) completed the study to 6 months. Twenty-three (70%) intervention participants were followed up at 12 months.

**Methods:**

All participants received normal care. Intervention participants attended facilitated meetings twice-weekly for 12 weeks, and followed a non-energy-restricted WFPB diet with vitamin B_12_ supplementation.

**Results:**

At 6 months, mean BMI reduction was greater with the WFPB diet compared with normal care (4.4 vs 0.4, difference: 3.9 kg m^−2^ (95% confidence interval (CI)±1), *P*<0.0001). Mean cholesterol reduction was greater with the WFPB diet, but the difference was not significant compared with normal care (0.71 vs 0.26, difference: 0.45 mmol l^−1^ (95% CI±0.54), *P*=0.1), unless dropouts were excluded (difference: 0.56 mmol l^−1^ (95% CI±0.54), *P*=0.05). Twelve-month mean reductions for the WFPB diet group were 4.2 (±0.8) kg m^−^^2^ BMI points and 0.55 (±0.54, *P*=0.05) mmol l^−1^ total cholesterol. No serious harms were reported.

**Conclusions:**

This programme led to significant improvements in BMI, cholesterol and other risk factors. To the best of our knowledge, this research has achieved greater weight loss at 6 and 12 months than any other trial that does not limit energy intake or mandate regular exercise.

## Introduction

### Background

Globally, the obesity epidemic worsens.^1, 2^ In 2014 more than 600 million adults were obese and a further 1.9 billion adults were overweight.^3^ In New Zealand, 31% of adults are obese and 35% are overweight.^4^ A raised body mass index (BMI) is associated with many forms of cancers; type 2 diabetes (T2DM); osteoarthritis; obstructive sleep apnoea; a shorter life expectancy; a lower quality of life and cardiovascular disease.^2, 3, 5, 6^ Additionally, these diseases impose a significant financial burden on both the health-care system and the wider economy.^7^

Many individuals attempt to lose weight by making changes to their diet, and commercial weight loss programmes are part of a multibillion-dollar market.^8^ Reviews of dietary interventions for weight loss fail to demonstrate superiority of one diet over another.^8, 9, 10^ In one review of 48 trials comparing commercial interventions, both low-carbohydrate and low-fat diet approaches were deemed effective at 6 months; participants lost on average 8 kg, with 1–2 kg regained at 12 months.^10^ Massive weight loss has also been achieved through a very high carbohydrate (⩾90 % dietary energy), calorie-restricted 'Rice Diet' as far back as 1940. This extremely restrictive approach has shown in one case series an average 63.9 (SD±17.2) kg weight loss for 106 patients.^11, 12^

The whole food plant-based (WFPB) diet is high in micronutrient density and the most frequently researched iterations are low in fat, which comprises approximately 7–15% of total energy.^13, 14, 15^ Interventions using the WFPB diet (alone, or with exercise and stress reduction) have demonstrated: reversal of ischaemic heart disease, improvements in glycaemic control, weight loss, long-term acceptability and sustainability, and reduction of prostate-specific antigen in biopsy-proven low-grade prostate cancer.^16, 17, 18, 19, 20, 21, 22^

Other nonclinical implications deserve attention. A WFPB diet generally requires less land, energy, and water than a diet high in animal products.^23^ On a per calorie basis, a high-meat diet (>100 g per day) produces 2.5 times more greenhouse gas emissions than a vegan diet.^24^ Farming the estimated 70 billion land animals consumed annually contributes between 14.5 and 51% of total human-induced greenhouse gas emissions, more than all of transportation.^23, 25, 26, 27, 28^

### Objective

We aimed to investigate the effectiveness of our community-based WFPB dietary programme in a population of New Zealanders. Our programme was unique because we focused on creating long-term behavioural changes through developing practical skills (especially cooking).^13, 16, 17, 19^

## Subjects and methods

### Study overview

The BROAD study was a prospective, two-arm, parallel, superiority study run from August 2014 to initially February 2015 (subsequently extended). We compared standard medical care (control) with standard medical care plus a diet change programme (intervention).

### Recruitment of participants

The intervention involved patients from a group general practice in Gisborne, the region with New Zealand's highest rates of socioeconomic deprivation, obesity and type 2 diabetes.^29, 30^ Inclusion criteria were age 35–70 and either obese (BMI⩾30 kg m^−2^) or overweight (BMI⩾25 kg m^−^^2^), with a diagnosis of one of type 2 diabetes, ischaemic heart disease, or the cardiovascular risk factors of hypertension or hypercholesterolaemia (for definitions see Table 1, baseline characteristics). Participant exclusion criteria were diagnoses of life-threatening comorbidities; thyroid disease; coronary artery bypass grafting within 6 weeks; myocardial infarction within 1 month; angioplasty within 6 months; >50% stenosis of the left main coronary artery; unresponsive congestive heart failure; malignant uncontrolled arrhythmias; homozygous hypercholesterolaemia; severe mental health disorders; current alcohol or drug misuse; currently smoking; currently pregnant or breastfeeding women, prior bariatric surgery, other conditions that directly affect weight (e.g. lead toxicity, malignancy).

### Enrolment

**Figure 1:**
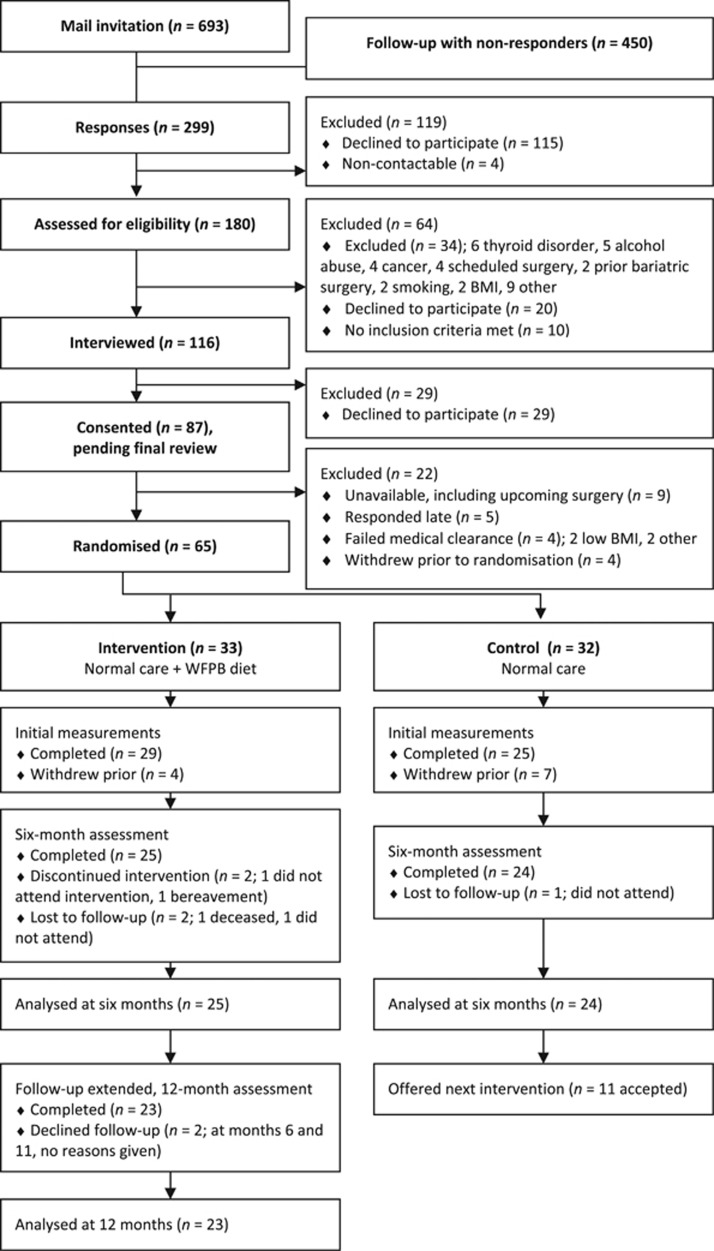
Patient flow.

We invited 693 candidates by letter in May 2014 and sent a reminder to 450 non-responders 1 month later. The letter included consent for EMR screening, general and nutritional self-efficacy questionnaires, a question on self-esteem, and three questions on readiness for change.^31, 32, 33^ Doctors reviewed the EMR and confirmed eligibility. The research coordinator conducted 116 interviews, and explained randomisation, the WFPB diet and showed examples of appropriate meals. Candidates listed the benefits and downsides of allocation to either trial group before providing written consent. We randomised 65 participants total (Figure 1).

### Intervention

Intervention participants followed a low-fat plant-based diet (approximately 7–15% total energy from fat).^13^ We chose a low-fat iteration of the plant-based diet as this has been shown with previous research to achieve optimal outcomes, especially for heart disease and weight loss.^13, 14, 17, 18^ This dietary approach included whole grains, legumes, vegetables and fruits. Participants were advised to eat until satiation. We placed no restriction on total energy intake. Participants were asked to not count calories. We provided a ‘traffic-light' diet chart to participants outlining which foods to consume, limit or avoid (Supplementary Table S1). We encouraged starches such as potatoes, sweet potato, bread, cereals and pasta to satisfy the appetite. Participants were asked to avoid refined oils (e.g. olive or coconut oil) and animal products (meat, fish, eggs and dairy products). We discouraged high-fat plant foods such as nuts and avocados, and highly processed foods. We encouraged participants to minimise sugar, salt and caffeinated beverages. We provided 50 μg daily vitamin B_12_ (methylcobalamin) supplements. The intervention group attended 2-h evening sessions twice-weekly for 12 weeks. We ran sessions at a local polytechnic, incorporating a chef-guided cooking tutorial and presentation by doctors, with a discussion. Programme outline provided (Supplementary Table S2). Special events included screening the documentary *'Forks Over Knives'* and an accompanying film endorsing the WFPB diet; discussion sessions; restaurant meals; quiz night; potlucks; and graduation ceremony. Both intervention and control group participants received $40 petrol vouchers to cover travel costs and received a birthday card along with a voucher redeemable for a native plant.

### Data collection and measurements

After enrolment was completed, both groups completed the same questionnaires. We used the Big Five Inventory (BFI 44) personality assessment for traits associated with adherence.^34, 35^ We used the Short Form 36 health survey version 2 (SF-36v2) to measure self-perceived health status. For diet and exercise, we used 3-day recall forms to track dietary indiscretions and exercise. We averaged daily exercise scores using participant reported exertion (Borg Rating of Perceived Exertion scale) multiplied by minutes of exercise.^36^ We asked four questions to assess food enjoyment, and calculated food spending by amount divided by frequency to get the cost per day. We counted regular medications from the EMR, excluding as needed (PRN) medications except for glyceryl trinitrate. We discussed prescribing with general practitioners, but general practitioners made all prescribing decisions. We used an online risk assessment (CVD RA) calculator to estimate the 5-year risk of a cardiovascular event.^37, 38^ We used the Behavior Change Technique Taxonomy to classify aspects of the intervention (Supplementary Table S3).^39^ The research coordinator performed measurements using the same clinic and equipment, with intervention and control measured 1 week apart. Participants removed shoes and outer clothing. We measured blood pressure (BP) after 5 min seated using the automated digital Pro 3400 BP device (Welch Allyn, Auckland, New Zealand) on the left arm. We then measured height, weight, waist circumference (WC) and a second BP reading. We measured height standing using a wall-mounted stadiometer and weighed participants using a calibrated medical scale (Wedderburn model WM202, Napier, New Zealand). We measured WC standing beside the participant, pulled snug at the height of the navel. T-Lab Gisborne conducted all blood testing. We asked participants to fast for 12 h prior. General chemistry and lipids were measured on the COBAS c6000 (Roche Diagnostics, Auckland, New Zealand), and HbA_1c_ on the D-10 (Bio-Rad Laboratories, Auckland, New Zealand).

### Outcomes

The primary outcomes were BMI and cholesterol. Secondary outcomes included changes in medication usage, quality of life, cardiovascular risk factors, cardiovascular events, or progression to surgery, and transfer to a higher level of care. The initial end point was 6 months. We measured personality inventory for factors associated with adherence, and collected data on any harms.

### Minor changes to trial design

At the 6-month end point, we observed significant differences between the intervention and control groups, and we offered the intervention to the control group. Ethics approval was obtained to extend follow-up to 3 years total, and the protocol was updated.

### Randomisation

We randomised participants in the order of interview by 1:1 sequence from random.org. The allocation was passed to another researcher who assigned groups. We randomised five married, partnered and related pairs together.

### Blinding

It was not feasible to blind participants. The researcher performing measurements was aware of allocation. The participants' usual health-care providers were not informed of allocation, although they could ask participants. The statistician was blinded.

### Sample size and analysis

We assumed an 80% chance of demonstrating an effect on cholesterol and BMI at a confidence level of 95% (*P*<0.05), based on previous research.^40^ Sample size calculation indicated 30 participants per group. Allowing for a 30% dropout rate, we sought 40 participants per group. However, due to time constraints we started with 33 intervention and 32 control group participants. All predefined outcomes were analysed using the pairwise deletion method. We additionally analysed a subgroup that excluded intervention dropouts for between-group differences in total cholesterol. All *t*-tests were two-tailed. Comparison of results between the two groups were analysed using unpaired *t*-test, after performing an *f*-test to determine whether the groups of results had variances that were not significantly different. One set of intergroup differences came close to an unequal variance; however, analysis on the basis of an unequal variance did not change the significance of the result. Statistics were analysed using an external blinded statistician, not otherwise involved in the study, using Stata (College Station, TX, USA).

## Results

### Losses and exclusions

During the research, one intervention participant died in a motor vehicle accident at week 19. One control group participant reportedly began a plant-based diet from week 6 and continued in the control group.

### Results

Of the 693 total people invited by mail, 65 (9.4%) were randomised to either intervention or control. At the 6-month assessment 49 of 65 (75.4%) participants were followed up: 25 (76%) of the intervention group and 24 (75%) of the control. From the intervention group 23 (70%) were followed up at 1 year (Figure 1). Baseline characteristics are available in Table 1.

### BMI end point

The primary outcome of BMI change was available for 25 (76%) participants in the intervention group and 24 (75%) in the control at 6 months. Individual BMI changes over time are shown in Supplementary Figure S1. Intervention and between-group reductions in BMI and weight were statistically significant at all measurement points (all *P*<0.0001, unless stated) (Tables 2 and 4). At 6 months mean intervention BMI reduction was 4.4 (range 0.4–7.4, 95% confidence interval (CI) 3.7–5.1) kg m^−^^2^, and at 12 months this was 4.2 (range 0.5–8.3, 95% CI 3.4–5) kg m^−^^2^. From 6 to 12 months intervention BMI increased non-significantly by 0.4 (range −1.3 to 4, 95% CI −0.1 to 0.9, *P*=0.12) kg m^−^^2^. For weight, intervention reduction at 6 months was 12.1 (range 1.4–27.3, 95% CI 9.9–14.3) kg, and at 12 months was 11.5 (range 1.6–28.3, 95% CI 9–14) kg. Within the control group, there were no significant reductions in BMI at 3 (*P*=0.2) or 6 months (*P*=0.18) (Table 3). At 6 months the between-group analyses showed differences of 3.9 (95% CI±1) kg m^−^^2^, and 10.6 (95% CI±2.9) kg, which favoured the intervention.

### Cholesterol end point

The primary outcome of cholesterol change was available for 25 (76%) participants in the intervention group and 23 (72%) in the control at 6 months. Individual cholesterol changes over time are shown in Supplementary Figure S2. Within the intervention group mean reduction in total cholesterol was statistically significant at all time periods, although there was a smaller effect size with time: at month 3 it was 0.95 (95% CI 0.51–1.39, *P*<0.001) mmol l^−1^; at month 6 it was 0.71 (95% CI 0.28 to 1.14, *P*<0.01) mmol l^−1^; and at month 12 it was 0.55 (95% CI 0.01–1.09, *P*=0.05) mmol l^−1^. In the control group we observed a statistically significant mean reduction in total cholesterol at month 3, at 0.28 (95% CI 0.05–0.52, *P*=0.03) mmol l^−1^, but this was not significant by month 6, at 0.26 (95% CI −0.10 to 0.62, *P*=0.15) mmol l^−1^. Comparing standard care plus dietary programme (intervention) to standard care (control) at month 6, our analysis showed a nonsignificant reduction in total cholesterol at 0.45 (95% CI −0.09 to 1.00, *P*=0.10). *Post hoc* subgroup analysis for total cholesterol difference excluding intervention group dropouts showed a significant reduction of 0.56 (95% CI±0.54, *P*=0.05) mmol l^−1^, which favoured the intervention. Statistically significant between-group HDL-cholesterol differences were seen from baseline to 6 months at 0.25 (95% CI±0.15, *P*<0.0001) mmol l^−1^.

### Secondary end points

Medication data were available at 6 months for 62 of 65 (95%) participants via the GP EMR. Control group medications increased from 74 to 80 over 6 months, an 8% increase, and intervention group medication usage decreased from 94 to 74 at 6 months, and to 67 over 12 months: a 29% decrease (Supplementary Table S4).

### Cardiovascular risk factors

The CVD RA within the intervention group decreased slightly from baseline to 3 months; 0.4% (95 CI±0.3, *P*=0.02), and the between-group difference was significant 0.6% (95% CI±0.4, *P*=0.02). There were no other significant differences observed for CVD RA. The HbA_1c_ between-group differences favoured the intervention, with a reduction of 5 (95% CI±3, *P*<0.001) mmol mol^−1^ at 6 months. From baseline to 12 months the intervention average HbA_1c_ reduced by 5 (range −1 to 15, 95% CI±2, *P*<0.0001) mmol mol^−1^. For the intervention group, two people with diabetes no longer met diagnostic criteria (i.e. HbA_1c_ ⩾50 mmol mol^−1^) at both 6 and 12 months. Higher starting HbA_1c_ correlated with a larger subsequent reduction to 3, 6 and 12 months (correlation tests, *P*<0.01 or less). Intervention WC reduced compared with baseline at all time periods (Table 2). No changes were seen in the control group (Table 3), and between-group differences demonstrated 10 (95% CI±4, *P*<0.0001) cm greater mean intervention reduction at 6 months. There were no transfers to higher-level care or acute admissions for cardiac-related care for any participant during the first 12 months of the research.

### Quality of life and other variables

Quality of life showed significant improvements in the intervention group for all measurement periods in both the ‘physical component summary' and the ‘mental component summary' (Table 2). The control group showed significant improvement to 6 months with the physical component summary (*P*=0.03) (Table 3). Between-group differences favouring intervention were significant at 6 months for both the physical component summary (*P*=0.03) and the mental component summary (*P*<0.01) (Table 4). At 6 months no significant between-group differences were seen for: average daily exercise, food enjoyment or food costs (Table 4). Statistically significant differences favouring the intervention were seen at 6 months for general self-efficacy (*P*=0.01), nutritional self-efficacy (*P*<0.0001) and self-esteem (*P*<0.01).

### Adherence

Average attendance for intervention evening sessions was 79%. Dietary indiscretions (diet) over 3 days were used as adherence measure, and intervention BMI change from 0 to 12 months was correlated with diet at 3, 6 and 12 months (correlation tests: *P*<0.0001, *P*=0.04, *P*<0.001, respectively). Baseline diet was not related to 12-month BMI change (correlation test, *P*=0.84). In the intervention group, indiscretions increased significantly from 1 (95% CI±1) at 3 months to 3 (±1) at 6 months (paired *t*-test; *P*<0.0001), and then increased significantly to 5 (±1) at 1 year (6–12 month increase paired *t*-test; *P*=0.001). Intervention adherence vs BMI reduction from 0 to 12 months is shown in Supplementary Figure S3. In the control group, we observed a significant decrease in dietary indiscretions from baseline to 6 months (Table 3), although these were much smaller than the intervention group (Table 2). In the control group there was no correlation between BMI change from baseline to 6 months and dietary indiscretions at 0, 3 or 6 months (correlation tests: *P*=0.38; 0.88 and 0.57, respectively).

### Harms

No serious harms relating to the intervention were reported. One intervention participant with a diagnosis of type 2 diabetes reported hypoglycaemia from week 1 consuming the WFPB diet, and his general practitioner reduced, and then later stopped his insulin. Two intervention participants developed low serum vitamin B_12_, which normalised with supplementation. At month 5, an intervention group participant underwent cholecystectomy for cholecystitis.

## Discussion

This randomised controlled trial compared a 12-week WFPB dietary programme to normal care alone. The intervention led to significant and sustained BMI and weight reduction at all measurement points compared with the control group. To the best of our knowledge, there are no randomised controlled trials that have achieved a greater average weight loss over a 6- or 12-month period, without mandating regular exercise or restricting total caloric intake.^9, 10, 41^ The key difference between this trial and other approaches to weight loss was that participants were informed to eat the WFPB diet *ad libitum* and to focus efforts on diet, rather than increasing exercise. The mechanism for this is likely the reduction in the energy density of the food consumed (lower fat, higher water and fibre).^42^ Multiple intervention participants stated 'not being hungry' was important in enabling adherence. Intervention participants were highly adherent with the dietary changes, although this decreased with time. Diet at 3 months correlated with weight loss at 12 months, but starting diet did not. These findings suggest an audited diet diary may be useful to predict success with a WFPB diet, and that those starting from a typical Western diet could expect similar results.

Our results show a reduction in cholesterol for the intervention group at all measurement points, and in the control at 3 months only. Between-group analysis showed statistically significant differences in cholesterol at 3 months, and at 6 months with subgroup analysis. The ability to detect differences is potentially reduced by intervention group reduction in medications, and a decrease in dietary adherence over time. HbA_1c_ reductions favoured the intervention and all intervention patients with a diabetes diagnosis improved while adherent, and two resolved their condition by HbA_1c_. At 6 months, the intervention compared with the control had an increased quality of life (SF-36v2), general and nutritional self-efficacy, and self-esteem, without significant changes in food enjoyment, cost or exercise. Total regular medication usage decreased in the intervention group (94 at baseline, 74 at 6 months, 67 at 12 months), and increased in the control group (74 at baseline, 80 at 6 months). CVD RA tools are widely used in New Zealand, and although we saw intervention WC, BMI and HbA_1c_ improve, the between-group CVD RA (which does not account for some of these) did not change significantly. Also, HDL-cholesterol tends to decrease on a plant-based diet, and previous research had shown this 'may not be helpful for predicting cardiovascular risk in individuals consuming a low-fat, plant-based diet'.^43^ Our analysis corroborates that this tool is not particularly appropriate for those consuming a WFPB diet.

Strengths of this research include randomisation, and the ‘real world' nature of the programme, which involved community-dwelling adults who were provided skills and education but were responsible for their own food choices. A previous survey identified a lack of information as the main barrier to beginning a WFPB diet, and information provision formed a large part of our programme.^44^ Factors promoting success in the intervention group included the 2-week preparation period, individualised feedback and rapid initial weight loss. Reports from intervention participants suggest family and acquaintances themselves benefitted from exposure to the WFPB diet.

Limitations include necessarily explaining the WFPB diet to all participants during informed consent, and perhaps as a consequence, we observed a significant improvement with control group dietary indiscretions. Increased testing for normal care participants may have led to more focused treatment, and the observed lipid reductions, and a reduction in effect size. In our research, 7 of 65 (11%) participants had a diagnosis of ischaemic heart disease, and we included participants without a necessarily elevated HbA_1c_ or cholesterol, so collectively these could have lessened effect size. As it stands, our average cholesterol reductions were not necessarily as large as those seen with previous research. Other research has also evaluated dietary records in more depth, or used a WFPB diet programme in combination with exercise or relaxation.^17, 19, 40^ Weaknesses for this research include that the intervention group was not perfectly adherent. The intervention cholesterol drop was largest at measurement points when adherence was highest, and as adherence decreased, cholesterol increased, suggesting a dose–response relationship. Consumption of ‘green' category foods was encouraged, but not monitored, although this could be of benefit. Our questions for exercise, food costs and dietary indiscretions involved self-reporting and recall, which could have introduced error. Another source for introducing inaccuracy was using EMR ethnicity data, which may have underrepresented rates of Māori participants. Finally, participant dropouts may have affected results, although they were roughly equal in both groups.

Our programme contrasts with other commonly used approaches: exercise and very-low-calorie diets, or bariatric surgery. Very-low-calorie diets have achieved equal or greater mean weight loss to that seen in our research.^45, 46^ However, medically supervised liquid ‘meal replacements' are not intended for ongoing use and are associated with 'high costs, high attrition rates, and a high probability of regaining 50% or more of lost weight in 1 to 2 years'.^47^ Increased risks include gallstones, cold intolerance, hair loss and constipation.^46^ This contrasts with our research, whereby many in the WFPB diet group improved in the 9 months following the 12-week intervention. The Cochrane review of bariatric surgery shows both greater and lesser reduction in BMI at 12 months compared with our results, although it tends to favour bariatric surgery.^48^ One recent study with Roux-en-Y gastric bypass showed an increased quality of life after bariatric surgery, but hospitalisation rates were 4–5 times higher than the comparison group.^49^ The Cochrane review states bariatric surgery studies tend to include young, 'low-risk', mainly female patients and that the 'longer-term impact of surgery on weight loss or comorbidities is unclear'. Because bariatric surgery candidates are motivated for change, they could also be suitable for a WFPB dietary programme. This approach poses no risk of surgical morbidity or mortality but does require more time with patients. A WFPB dietary programme can be utilised in centres where surgery is unavailable, and we estimate cost per patient to be substantially less than surgery.

Reviews comparing the WFPB approach to other diets show similar weight loss at 12 months for low-carbohydrate and low-fat diet approaches.^10, 50^ Individual studies that combine regular exercise with either unrestricted or energy-restricted low-carbohydrate diets have observed similar weight loss to our intervention at 6 months: 12.0 (95% CI±1.8) kg;^51^ and at 1 year: 10.9 (95% CI±1.2) kg,^52^ and 12.2 (includes telemonitoring, 95% CI±1.3) kg reductions.^53^ However, studies on the effects of low-carbohydrate diets have shown higher rates of all-cause mortality,^54^ decreased peripheral flow-mediated dilation,^55^ worsening of coronary artery disease,^56^ and increased rates of constipation, headache, halitosis, muscle cramps, general weakness and rash.^10^ Other energy-restricted diets can be effective for weight loss, for example, one study using a 1 500 kcal daily intake, with 50% carbohydrate, 30% fat and 20% protein achieved an average 17.3 (95% CI±1.6) kg reduction in weight at 36 weeks, which is significantly more than our results.^57^ However, by restricting amount of food eaten, patients are likely to feel hungry, and hunger scores have been shown to predict those at risk of weight regain.^58^ Low-fat interventions that encourage regular exercise have shown equal weight loss at 1 year with energy-restricted and non-energy-restricted approaches: 10.8 kg (95% CI not available and±1.6, respectively).^17, 52^

Generalising our results to the community, we felt there were several key differences. Our study population had a higher number of females and a higher mean age. Our rates of Māori participants were less than the Gisborne community and similar to the national average. We would estimate our participants to have a higher than average health literacy. To provide longer-term data, we have extended follow-up to observe the intervention group for 3 years total.

## Conclusion

Many patients are interested in making dietary changes, and the WFPB diet can be offered as a safe and effective option for losing weight and obtaining some reduction in cholesterol, without necessarily increasing exercise. The main advantage is in eating to satiation without restricting the amount of food eaten. This small study also showed several improvements with chronic disease risk factors and quality of life, which were largely maintained to 12 months. Future research could identify participants who are currently likely to succeed with a diet change, which could reduce dropout rates and increase effectiveness. Given the low cost of this intervention and the relative benefits of this dietary approach, this could be offered by policy makers and practitioners as promoting weight loss, and suitable for consumption in hospitals.

## Figures and Tables

**Table 1:** Participant characteristics at baseline according to study arm *(n=65)*

*Characteristic*	*Intervention (*n=*33)*	*Control (*n=*32)*
*Sex, n (%)*		
Female	22 (67)	17 (53)
Male	11 (33)	15 (47)
		
Age, years (±SD)	56±9.9	56±9.5

*Ethnicity*,^a^ *n (%)*		
NZ European	30 (90.9)	21 (65.6)
Māori	3 (9.1)	5 (15.6)
Other		6 (18.7)
		
*Diagnoses*^a^		
BMI^b^ (kg m^−^^2^)		
Obesity, ⩾30	29 (88)	30 (94)
Overweight, 25–29.9	4 (12)	2 (6)
Type 2 diabetes mellitus^c^	7 (21)	2 (6)
Cardiovascular pathology	20 (61)	20 (63)
Ischaemic heart disease^d^	4 (12)	3 (9)
Hypertension^e^	19 (58)	17 (52)
Hypercholesterolaemia^f^	17 (52)	15 (47)
		
*Clinical measures—values are means (95% CI)*		
Weight (kg)	94.8 (6.4)	96.9 (7.4)
BMI (kg m^−^^2^)	34.5 (1.6)	34.2 (2.3)
		
Cholesterol^g^ (mmol l^−1^)		
Total	5.4 (0.5)	5.5 (0.6)
Triglycerides	1.6 (0.3)	1.4 (0.3)
LDL	3.4 (0.4)	3.5 (0.5)
HDL	1.3 (0.1)	1.4 (0.2)
		
Waist circumference (cm)	108 (4)	110 (5)
Systolic BP (mm Hg)	133 (6)	132 (7)
Diastolic BP (mm Hg)	81 (3)	78 (3)
HbA_1c_ (mmol mol^−1^)	42 (3)	37 (2)
CVD RA (% 5-year risk)^h^	11.7 (2.5)	12.2 (1.9)
		
*Questionnaires*^i^		
SF-36 Physical component summary	47 (3)	49 (3)
SF-36 Mental component summary	52 (2)	54 (3)
Dietary indiscretions	26 (3)	22 (4)
Exercise	20 (9)	47 (23)
Food enjoyment	12 (1)	13 (1)
Food cost	23 (5)	22 (3)
General self-efficacy	32 (1)	32 (2)
Nutritional self-efficacy	15 (1)	16 (1)
Self-esteem	2.4 (0.3)	2.3 (0.3)
		
*Big Five Inventory Multidimensional Personality Assessment*		
Extraversion	3.1 (0.3)	3.3 (0.3)
Agreeableness	4 (0.2)	4 (0.3)
Conscientiousness	3.9 (0.2)	4.2 (0.2)
Neuroticism	2.7 (0.2)	2.4 (0.3)
Openness	3.6 (0.3)	3.5 (0.3)
		
*Readiness for change*		
Question 1	3.3 (0.2)	3.4 (0.2)
Question 2	3.3 (0.2)	3.3 (0.2)
Question 3	3.4 (0.2)	3.3 (0.2)

a From information on electronic medical records (EMR) system.

b Body mass index is calculated as the weight in kilograms divided by the square of the height in metres.

c Type 2 diabetes mellitus was defined by the New Zealand standard, HbA_1c_ ⩾50mmol mol^−1^ cutoff.

d Ischaemic heart disease included prior coronary stenting; prior coronary artery bypass grafting (CABG); prior cardiovascular ischaemic event; or antianginal medication use.

e Hypertension included pre-treatment BP>140/90 mm Hg or current antihypertensive medication prescription.

f Hypercholesterolaemia included those with pre-treatment total cholesterol >6mmol l^−1^ or current cholesterol-lowering medication prescription.

g To convert values for total, low-density lipoprotein cholesterol (LDL) and high-density lipoprotein cholesterol (HDL) from mmol l^−1^ to mg dl^−1^, multiply by 38.67, for triglycerides multiply mmol l^−1^ by 88.57.

h The cardiovascular disease risk calculator estimates the risk of a cardiovascular event occurring within a 5-year period, using age, current HbA_1c_, duration of type 2 diabetes, sex, smoking status, ethnicity, total cholesterol to HDL-cholesterol ratio, systolic BP and status regarding use of BP lowering medication.

i We used the Short Form 36 Health Survey (SF-36) for quality of life assessment and responses were scored using Optum Scoring Software v4.5. For 3-day food recall each ‘red category' or ambiguous food item scored one point, e.g. pasta with meat (1) and cheese (1)=2, values reflect total over 3 days. Exercise units were average for 3 days using minutes of exercise × rated perceived exertion, where value shown is in hundreds, that is, 32 in table=3200. Food enjoyment, self-efficacy and readiness for change used a 1–4 Likert scale, with 4 being highest. Pooled answers had maximum score: 16 for food enjoyment, 40 for general self-efficacy and 20 for nutritional self-efficacy. Self-esteem was a single question using a 1–5 Likert scale, with 1 as highest.

**Table 2:** Intervention group differences in outcomes at 3 (programme end), 6 and 12 months, *n*=*33* a

*Measurement time (months)*	*Mean value*				*Within-group difference*			P*-value*		
	*Baseline*	*3*	*6*	*12*	*0–3*	*0–6*	*0–12*	*0–3*	*0–6*	*0–12*
*Clinical measures*										
Weight (kg)	94.8 (6.4)	86.7 (6)	82.9 (6.1)	82.7 (6.8)	−8.6 (1.2)****	−12.1 (2.2)****	−11.5 (2.5)****	<0.0001	<0.0001	<0.0001
BMI (kg m^−^^2^)	34.5 (1.6)	31.5 (1.7)	30.2 (1.6)	30.2 (1.7)	−3.1 (0.3)****	−4.4 (0.7)****	−4.2 (0.8)****	<0.0001	<0.0001	<0.0001
Cholesterol *(mmol l*^−1^*)*	5.4 (0.5)	4.5 (0.4)	4.7 (0.4)	5 (0.6)	−1 (0.4)***	−0.7 (0.4)**	−0.5 (0.5)*	<0.001	<0.01	0.05
Triglycerides	1.6 (0.3)	1.8 (0.4)	1.9 (0.5)	1.8 (0.5)	0.2 (0.3)	0.3 (0.3)*	0.3 (0.5)	0.18	0.05	0.24
LDL	3.4 (0.4)	2.5 (0.3)	2.6 (0.3)	3 (0.5)	−0.9 (0.4)****	−0.8 (0.3)***	−0.6 (0.4)*	<0.0001	<0.001	0.01
HDL	1.3 (0.1)	1.1 (0.1)	1.2 (0.1)	1.3 (0.1)	−0.2 (0.1)****	−0.2 (0.1)**	−0.1 (0.2)	<0.0001	<0.01	0.38
Total:HDL (ratio)	4.3 (0.4)	4.6 (0.5)	4.6 (1.1)	3.9 (0.6)	0.1 (0.5)	0.3 (1)	−0.3 (0.5)	0.67	0.49	0.33
Waist circumference (cm)	108 (4)	101 (4)	98 (4)	99 (4)	−7 (2)****	−10 (2)****	−9 (3)****	<0.0001	<0.0001	<0.0001
Systolic BP (mm Hg)	133 (6)	130 (5)	132 (6)	142 (8)	-4 (6)	−2 (6)	7 (8)	0.19	0.44	0.06
Diastolic BP (mm Hg)	81 (3)	81 (3)	82 (3)	83 (4)	−1 (3)	0 (3)	1 (4)	0.67	0.79	0.51
HbA_1c_ (mmol mol^−1^)	42 (3)	38 (3)	39 (3)	37 (4)	−4 (2)****	−3 (2)**	−5 (2)****	<0.0001	<0.01	<0.0001
Creatinine *(*μmol l^−1^*)*	73 (6)	66 (5)	63 (5)	64 (6)	−7 (3)***	−11 (4)****	−9 (4)****	<0.001	<0.0001	<0.0001
CVD RA (% 5-year risk)	11.7 (2.5)	11.1 (2.4)	11.2 (2.4)	11.7 (2.8)	−0.4 (0.3)*	−0.3 (0.4)	0 (0.4)	0.02	0.08	0.85
										
*Questionnaires*										
SF-36 Physical component summary	47 (3)	55 (2)	55 (2)	55 (2)	7 (3)***	7 (3)****	8 (3)***	<0.001	<0.0001	<0.001
SF-36 Mental component summary	52 (2)	55 (2)	56 (2)	54 (2)	4 (2)**	4 (3)**	3 (3)*	<0.01	<0.01	0.02
Dietary indiscretions	26 (3)	1 (1)	3 (1)	5 (1)	−26 (4)****	−24 (3)****	−22 (4)****	<0.0001	<0.0001	<0.0001
Exercise	20 (9)	35 (13)	23 (10)	32 (18)	14 (15)	3 (12)	13 (20)	0.06	0.64	0.18
Food enjoyment	12 (1)	13 (1)	13 (1)	13 (1)	0 (1)	1 (1)*	1 (1)	0.43	0.02	0.14
Food cost	23 (5)	24 (6)	22 (4)	20 (6)	2 (8)	−1 (4)	−3 (5)	0.53	0.54	0.19
General self-efficacy	32 (1)	33 (3)	34 (1)	34 (2)	1 (3)	2 (2)*	2 (2)*	0.53	0.03	0.05
Nutritional self-efficacy	15 (1)	17 (1)	17 (1)	16 (1)	2 (1)***	2 (1)**	1 (1)*	<0.001	<0.01	0.05
Self-esteem	2.4 (0.3)	2.1 (0.4)	2 (0.3)	2.1 (0.4)	−0.3 (0.4)	−0.4 (0.4)*	−0.2 (0.5)	0.09	0.04	0.31

Abbreviations: HDL, high-density lipoprotein cholesterol; LDL, low-density lipoprotein cholesterol.

a Values are means (95% CI). Paired, two-tailed *t*-tests were performed for within-group comparisons; ****P<0.0001, ***P<0.001, **P<0.01, *P<0.05.

**Table 3:** Control group differences in outcome at 3 and 6 months, *n*=*32* a

*Measurement time (months)*	*Mean value*			*Within-group difference*		P-*value*	
	*Baseline*	*3*	*6*	*0 –3*	*0–6*	*0–3*	*0–6*
*Clinical measures*							
Weight (kg)	96.9 (7.4)	95.3 (7.7)	94.1 (7)	−1.2 (1.7)	−1.6 (2.1)	0.16	0.13
BMI (kg m^−2^)	34.2 (2.3)	33.5 (2.4)	33.2 (2.2)	−0.4 (0.6)	−0.5 (0.8)	0.2	0.18
Cholesterol (mmol l^−1^)	5.5 (0.6)	5.3 (0.8)	5.2 (0.7)	−0.3 (0.2)*	−0.3 (0.4)	0.02	0.15
Triglycerides	1.4 (0.3)	1.3 (0.5)	1.5 (0.5)	0 (0.2)	0.1 (0.3)	0.88	0.43
* *LDL	3.5 (0.5)	3.1 (0.7)	3.1 (0.6)	−0.5 (0.4)**	−0.4 (0.3)*	<0.01	0.02
HDL	1.4 (0.2)	1.5 (0.3)	1.5 (0.2)	0.1 (0.1)	0.1 (0.1)	0.07	0.11
Total:HDL (ratio)	4.2 (0.5)	4.1 (1)	4.2 (0.8)	−0.3 (0.4)	−0.2 (0.4)	0.13	0.21
Waist circumference (cm)	110 (5)	110 (5)	110 (5)	−1 (2)	0 (3)	0.37	0.96
Systolic BP (mm Hg)	132 (7)	131 (6)	127 (7)	−1 (7)	−4 (7)	0.7	0.21
Diastolic BP (mm Hg)	78 (3)	81 (3)	78 (3)	2 (3)	0 (3)	0.13	0.99
HbA_1c_ (mmol mol^−1^)	37 (2)	39 (3)	39 (3)	2 (1)*	2 (1)**	0.01	<0.01
Creatinine (μmol l^−1^)	75 (4)	73 (6)	72 (6)	−1 (3)	−2 (4)	0.35	0.32
CVD RA (% 5-year risk)	12.2 (1.9)	12 (2.4)	12.2 (2.1)	0.1 (0.3)	0.2 (0.4)	0.42	0.38
							
*Questionnaires*							
SF-36 Physical component summary	49 (3)	50 (4)	52 (3)	0 (3)	3 (2)*	0.77	0.03
SF-36 Mental component summary	54 (3)	55 (4)	53 (3)	0 (4)	−2 (3)	0.97	0.33
Dietary indiscretions	22 (4)	19 (3)	18 (4)	−3 (4)	−4 (3)*	0.1	0.01
Exercise	47 (23)	39 (16)	36 (24)	−1 (26)	−9 (17)	0.93	0.26
Food enjoyment	13 (1)	13 (1)	13 (1)	0 (1)	0 (1)	0.25	0.62
Food cost	22 (3)	24 (5)	19 (5)	1 (6)	−5 (5)*	0.84	0.03
General self-efficacy	32 (2)	31 (3)	30 (3)	−1 (2)	−1 (2)	0.25	0.19
Nutritional self-efficacy	16 (1)	14 (1)	14 (1)	−2 (1)***	−2 (1)*	<0.001	0.01
Self-esteem	2.3 (0.3)	2.3 (0.3)	2.5 (0.4)	0.2 (0.3)	0.3 (0.3)*	0.16	0.04

Abbreviations: HDL, high-density lipoprotein cholesterol; LDL, low-density lipoprotein cholesterol.

a Values are presented as means (95% CI). Paired, two-tailed *t*-tests were performed for within-group comparisons; ****P<0.0001, ***P<0.001, **P<0.01, *P<0.05.

**Table 4:** Differences in outcomes between intervention and control groups at 3 and 6 months^a^

*Measurement time (months)*	*Differences in change, mean*		P*-value*	
	*0–3*	*0–6*	*0–3*	*0–6*
*Clinical measures*				
Weight (kg)	−7.5 (2) ****	−10.6 (2.9) ****	<0.0001	<0.0001
BMI (kg m^−2^)	−2.7 (0.7) ****	−3.9 (1) ****	<0.0001	<0.0001
Cholesterol (mmol l^−1^)	−0.7 (0.5) *	−0.5 (0.5)	0.01	0.10
Triglycerides	0.2 (0.4)	0.2 (0.5)	0.29	0.32
LDL	−0.4 (0.5)	−0.4 (0.5)	0.15	0.12
HDL	−0.3 (0.1) ****	−0.2 (0.1) ****	<0.0001	0.001
Total:HDL (ratio)	0.4 (0.6)	0.6 (1.1)	0.18	0.28
Waist circumference (cm)	−7 (2) ****	−10 (4) ***	<0.0001	<0.0001
Systolic BP (mm Hg)	−2 (9)	2 (8)	0.57	0.63
Diastolic BP (mm Hg)	−3 (4)	0 (4)	0.18	0.84
HbA_1c_ (mmol mol^−1^)	−6 (2) ****	−5 (3) ****	<0.0001	<0.001
Creatinine (μmol l^−1^)	−5 (4) *	−9 (6) **	0.01	<0.01
CVD RA (% 5-year risk)	−0.6 (0.4) *	−0.5 (0.5)	0.02	0.06
				
*Questionnaires*				
SF-36 Physical component summary	6 (4) **	4 (4) *	<0.01	0.03
SF-36 Mental component summary	4 (4)	6 (4) **	0.08	<0.01
Dietary indiscretions	−23 (5) ****	−20 (4) ****	<0.0001	<0.0001
Exercise	15 (29)	12 (20)	0.29	0.23
Food enjoyment	0 (1)	1 (1)	0.90	0.26
Food cost	2 (9)	4 (6)	0.69	0.21
General self-efficacy	2 (4)	3 (3) *	0.23	0.01
Nutritional self-efficacy	4 (1) ****	4 (2) ****	<0.0001	<0.0001
Self-esteem	−0.5 (0.4) *	−0.8 (0.5) **	0.03	<0.01

a Values are presented as means (95% CI). Unpaired *t*-tests were performed for between-group differences (*P*-values). Asterisks for *P*-values; ****P<0.0001, ***P<0.001, **P<0.01, *P<0.05. Calculated as (differences in intervention compared with baseline)–(differences in control compared with baseline), i.e. negative difference represent greater intervention reduction compared with control. Negative scores for dietary indiscretions and self-esteem favour intervention. Positive scores for SF-36 summaries, exercise, food enjoyment, general self-efficacy and nutritional self-efficacy favour intervention. Abbreviations: HDL, high-density lipoprotein cholesterol; LDL, low-density lipoprotein cholesterol.
