# Supplementary figures and images for: The BROAD study: A randomised controlled trial using a whole food plant-based diet in the community for obesity, ischaemic heart disease or diabetes
Source: Nutr Diabetes. 2017 Mar 20;7(3):e256–. doi: 10.1038/nutd.2017.3 (PMC5380896; doi:10.1038/nutd.2017.3)

**Intervention group BMI**

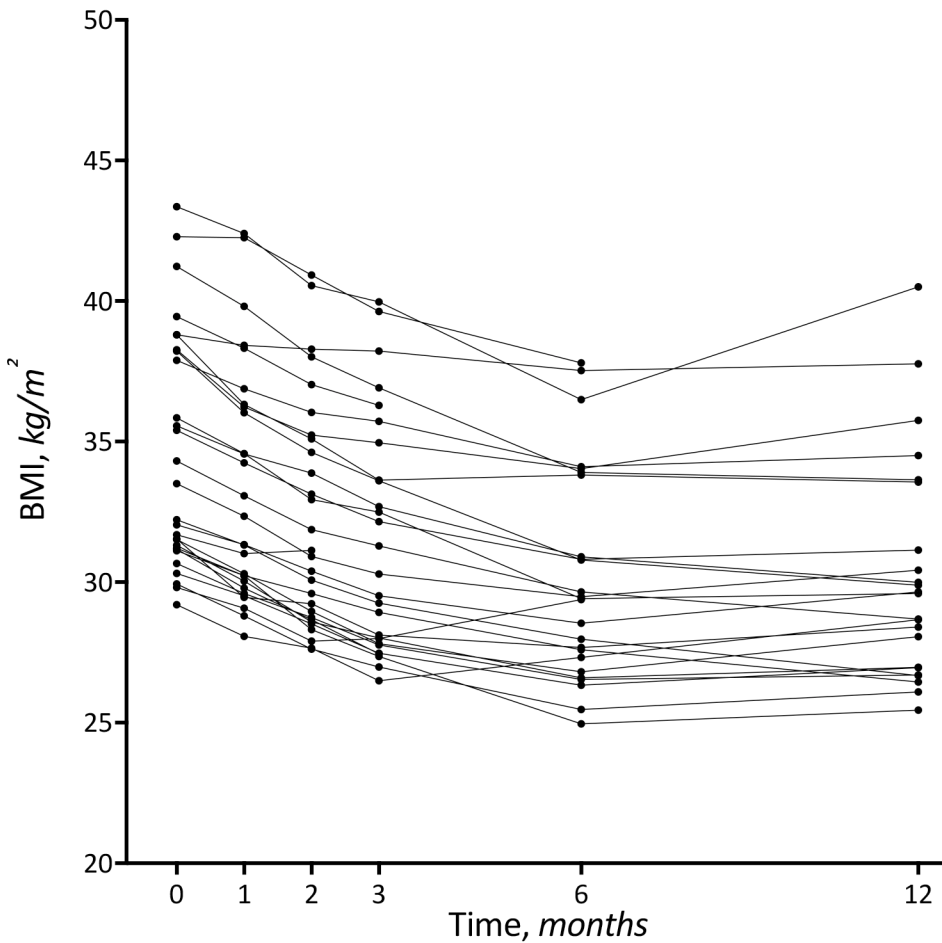

**Control group BMI**

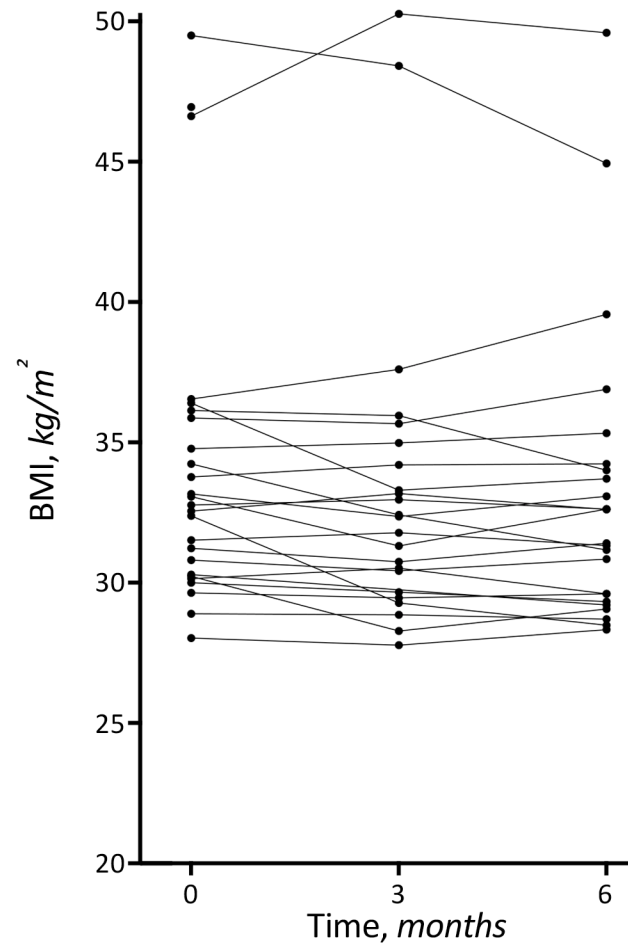

Supplement: Supplementary Figure 1 [file nutd20173x1.pdf]

**Intervention group total cholesterol**

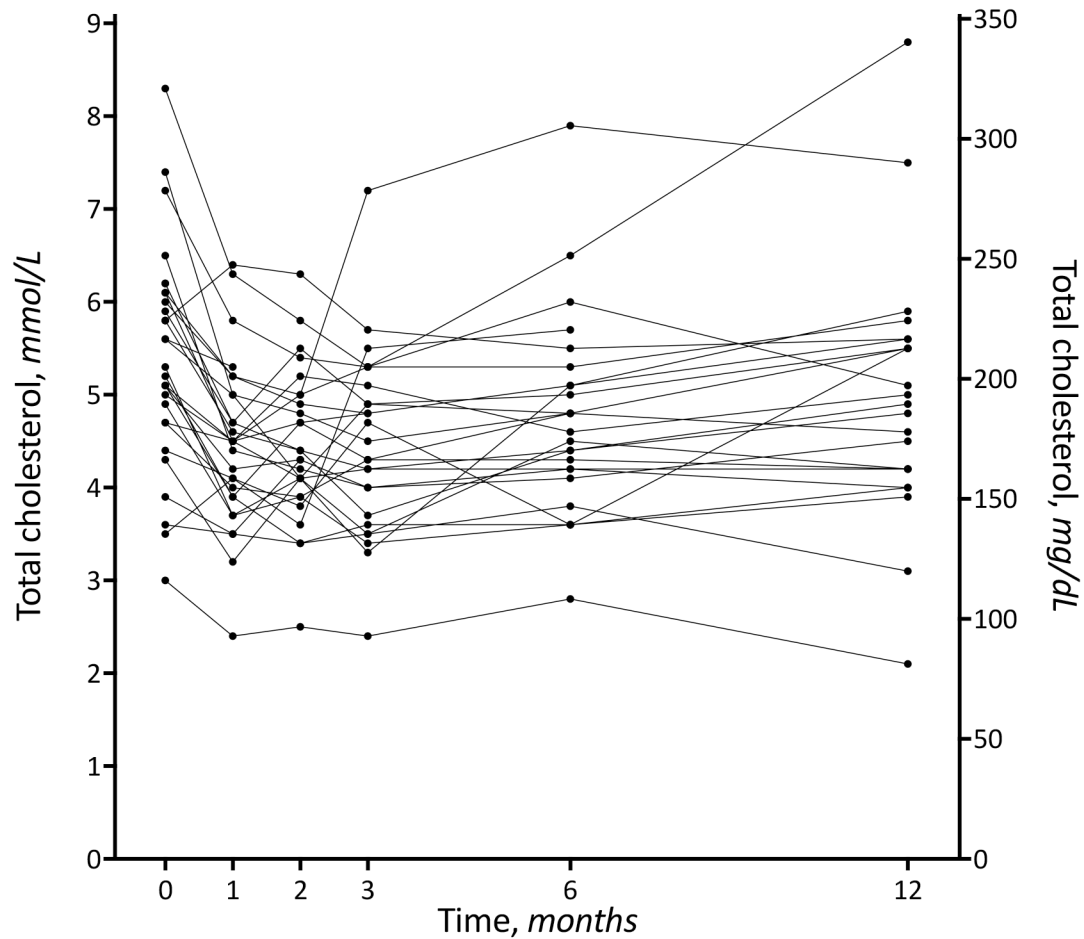

**Control group total cholesterol**

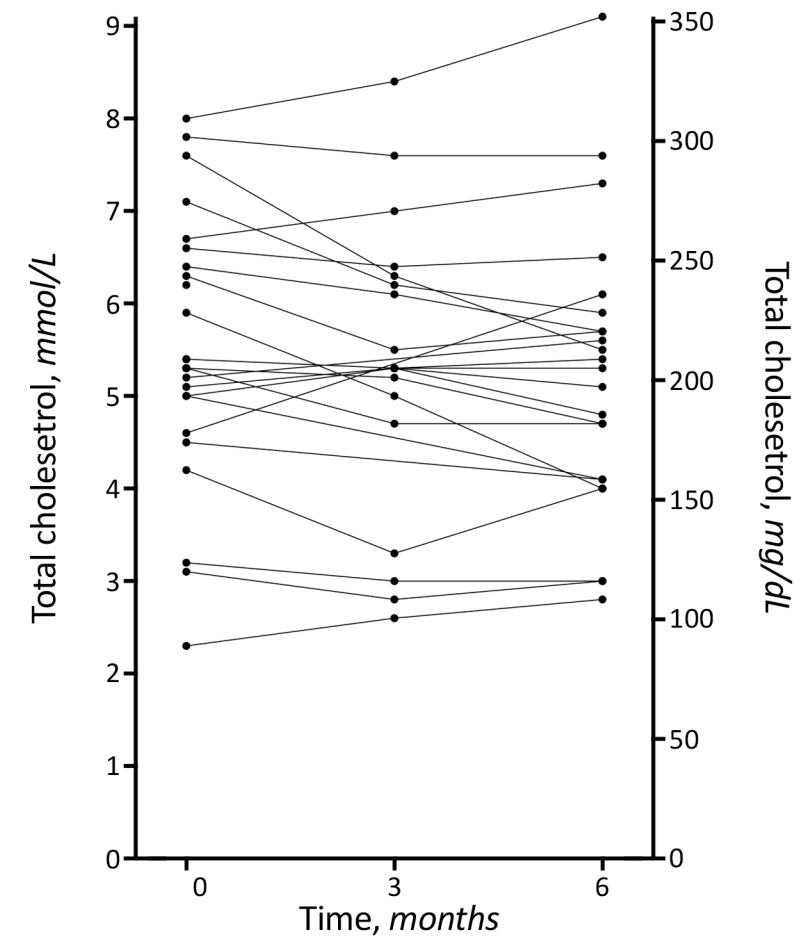

Supplement: Supplementary Figure 2 [file nutd20173x2.pdf]
