# Supplementary material for: The BROAD study: A randomised controlled trial using a whole food plant-based diet in the community for obesity, ischaemic heart disease or diabetes
Source: Nutr Diabetes. 2017 Mar 20;7(3):e256–. doi: 10.1038/nutd.2017.3 (PMC5380896; doi:10.1038/nutd.2017.3)
Supplement: Supplementary Figure 3 [file nutd20173x3.pdf]

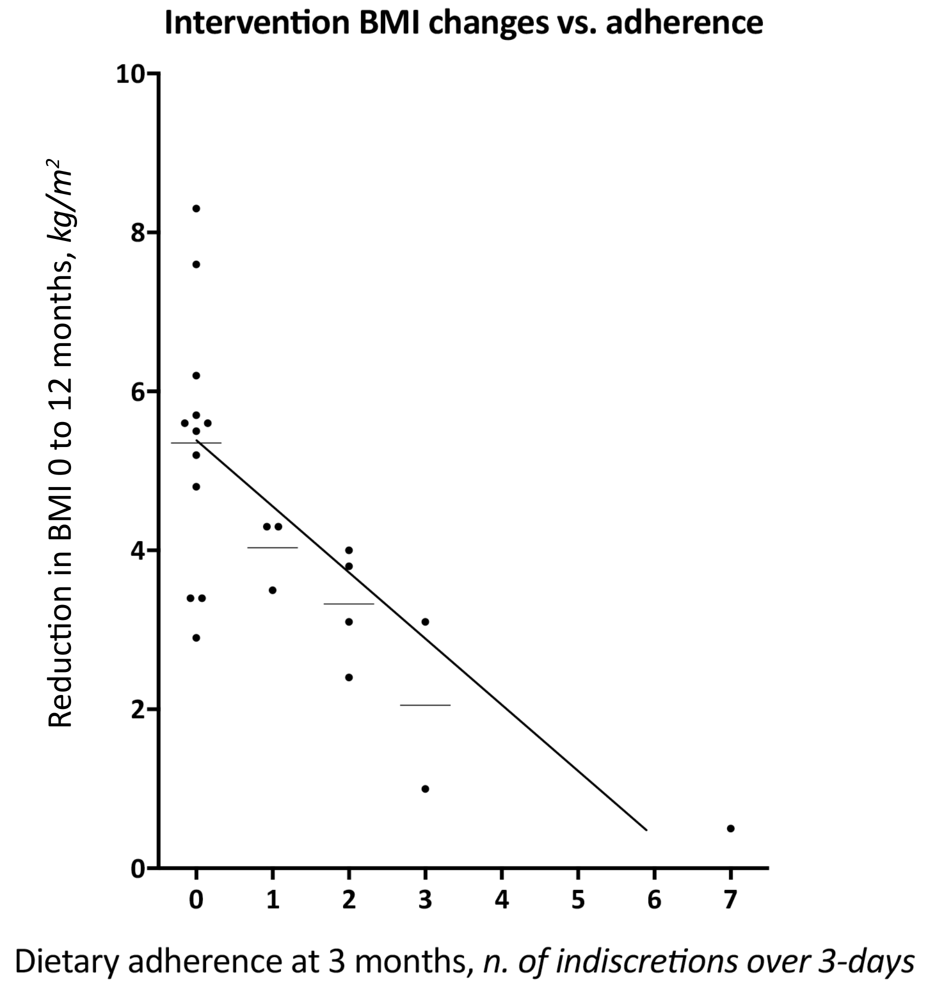
Intervention BMI changes vs. adherence
Reduction in BMI 0 to 12 months, kg/m2
Dietary adherence at 3 months, n. of indiscretions over 3-days
0
2
4
6
8
10
0
1
2
3
4
5
6
7
