# Supplementary material for: The BROAD study: A randomised controlled trial using a whole food plant-based diet in the community for obesity, ischaemic heart disease or diabetes
Source: Nutr Diabetes. 2017 Mar 20;7(3):e256–. doi: 10.1038/nutd.2017.3 (PMC5380896; doi:10.1038/nutd.2017.3)
Supplement: Supplementary Table 1 [file nutd20173x4.docx]

| **Supp. Table 1.** Total medication usage for both groups as per EMR^a^ | | | | |
| --- | --- | --- | --- | --- |
|  | Baseline | 3m | 6m | 12m |
| Control (*n*) | *32* | *32* | *32* | - |
| Total medications | 74 | 82 | 80 | - |
|  |  |  |  |  |
| Intervention (*n*) | *33* | *33* | *33* | *33* |
| Total medications | 94 | 74 | 74 | 67 |

^a^Data for 65 of 65 participants, however, medications carried forward from last measurement for one intervention participant and two control participants for whom data was not available. Medication number does not include vitamin B12 supplements.
