# Supplementary material for: The BROAD study: A randomised controlled trial using a whole food plant-based diet in the community for obesity, ischaemic heart disease or diabetes
Source: Nutr Diabetes. 2017 Mar 20;7(3):e256–. doi: 10.1038/nutd.2017.3 (PMC5380896; doi:10.1038/nutd.2017.3)
Supplement: Supplementary Table 2 [file nutd20173x5.docx]

| **Supp. Table 2.** Traffic light diet reference chart supplied to participants^†^ | | | |
| --- | --- | --- | --- |
| **Ultra-green** | **Green: Eat Every Day!** | **Orange: Use Sparingly, If At All** | **Red: Set Aside, Do Not Use** |
| These are the most nutrient dense and low energy foods available   - Spinach - Silverbeet - Kale - Bok Choy - Broccoli - Beetroot greens - Watercress - Herbs and spices | - Vegetables - Fruit - Whole Grains - Sauces and seasonings (without added oil) | - Salt - Sugar and other sweeteners - Processed flour - Tempeh and Tofu - Soy Milk, Almond Milk, Oat Milk (without added oils) - Caffeine and alcohol | - Poultry, Fish, Meat, Eggs - Dairy Products - Oils   Strongly limit due to fat content:   - Nuts and Seeds, Tahini - Avocado and Coconut, - Coconut Milk |

^†^Tempeh and Tofu were chosen as orange category foods due to their high fat content. Nuts and seeds were chosen as red category foods mainly due to high fat content. Participants were advised up to 2 tablespoons daily of Flaxseeds or Chia seeds can be consumed, if desired.
