# Supplementary material for: The BROAD study: A randomised controlled trial using a whole food plant-based diet in the community for obesity, ischaemic heart disease or diabetes
Source: Nutr Diabetes. 2017 Mar 20;7(3):e256–. doi: 10.1038/nutd.2017.3 (PMC5380896; doi:10.1038/nutd.2017.3)
Supplement: Supplementary Table 3 [file nutd20173x6.docx]

| **Supp. Table 3.** Behaviour Change Technique Taxonomy^a^ |
| --- |
| **Goals and planning**  1·1 Goal setting (behavior) ++  1·2 Problem solving ++  1·3 Goal setting (outcome) ++  1·4 Action planning ++  1·5 Review behavior goal(s) ++  1·6 Discrepancy between current behavior and goal ++  1·7 Review outcome goal(s) ++ |
| **Feedback and monitoring**  2·1 Monitoring of behavior by others without feedback ++  2·2 Feedback on behaviour ++  2·3 Self-monitoring of behaviour ++  2·6 Biofeedback ++  2·7 Feedback on outcome(s) of behavior ++ |
| **Social support**  3·1 Social support (unspecified) +  3·2 Social support (practical) ++  3·3 Social support (emotional) ++ |
| Shaping knowledge  4·1 Instruction on how to perform the behavior ++  4·2 Information about Antecedents +  4·3 Re-attribution +  4·4 Behavioral experiments + |
| **Natural consequences**  5·1 Information about health consequences ++  5·2 Salience of consequences +  5·3 Information about social / environmental consequences ++  5·6 Information about emotional consequences + |
| **Comparison of behaviour**  6·1 Demonstration of the behavior ++  6·2 Social comparison +  6·3 Information about others’ approval + |
| **Associations**  7·4 Remove access to the reward + |

| **Repetition and substitution**  8·1 Behavioral practice/rehearsal ++  8·2 Behavior substitution ++  8·4 Habit reversal ++  8·6 Generalisation of target behavior ++ |
| --- |
| **Comparison of outcomes**  9·1 Credible source ++  9·2 Pros and cons ++  9·3 Comparative imagining of future outcomes ++ |
| **Reward and threat**  10·1 Material incentive (behavior) ++  10·2 Material reward (behavior) ++  10·3 Non-specific reward +  10·4 Social reward ++  10·7 Self-incentive ++  10·9 Self-reward ++  10·10 Reward (outcome) ++  10·11 Future punishment + |
| **Regulation**  11·2 Reduce negative emotions ++  11·3 Conserving mental resources ++ |
| Antecedents  12·1 Restructuring the physical environment ++  12·2 Restructuring the social environment +  12·3 Avoidance/reducing exposure to cues for the behavior + |
| **Identity**  13·1 Identification of self as role model ++  13·2 Framing/reframing ++  13·3 Incompatible beliefs +  13·4 Valued self-identify +  13·5 Identity associated with changed behavior + |
| **Scheduled consequences**  14·8 Reward alternative behavior + |
| **Self-belief**  15·1 Verbal persuasion about capability ++  16·3 Vicarious consequences ++ |

^a^Two researchers classified the intervention using the Behavior Change Technique Taxonomy, which is an extensive taxonomy of consensually agreed, distinct behavioural change techniques that enables ease of comparison with other research, and easier replication of research. ++ is strongly present, + is present. Discrepancies were resolved by consensus.
