# Supplementary material for: The BROAD study: A randomised controlled trial using a whole food plant-based diet in the community for obesity, ischaemic heart disease or diabetes
Source: Nutr Diabetes. 2017 Mar 20;7(3):e256–. doi: 10.1038/nutd.2017.3 (PMC5380896; doi:10.1038/nutd.2017.3)
Supplement: Supplementary Table 4 [file nutd20173x7.docx]

| **Supp. Table 4.** Programme outline for intervention participants | | |
| --- | --- | --- |
| Week | Information on session or intervention component | Foods cooked and skills practiced |
| *Introduction period – participants asked to start making changes, or preparing for WFPB diet* | | |
| 1 | Doctors held individual patient consultations. | |
|  | Cinema opening night. Participants given “*Forks Over Knives*” DVD, vitamin B12 supplement, a card for 20% discount at a local bakery and restaurant, and a list of restaurants that serve WFPB options. | Hors d’oeuvre. |
|  | Programme outline. Introduction to the WFPB diet. | Blind-taste test for 10 non-dairy milk alternatives. |
| 2 | Strategies to keep friends, family, and colleagues supportive of lifestyle change. Credit: Dr Doug Lisle. | Demonstration: hummus and frozen banana ‘ice-cream’. |
|  | SMART goal setting, and photo supermarket tour. Participants given “*The Starch Solution*” book. | Acceptable prepared and packaged foods demonstrated. |
| *Participants asked to begin WFPB diet* | | |
| 3 | Measurement and blood test results, explained test interpretation. | Cooked oatmeal, and pumpkin pancakes. |
|  | Salt, Sugar, and Spices. | Pumpkin and kumara, and red lentil soups. Displayed herbs and spices. |
| 4 | Packaged foods, how to calculate percentage energy from fat. | Made pizzas, including the bases. |
|  | Approaches for travel, restaurants, socialising. Practiced ordering off menus from local restaurants. | Oil-free dips and curry pastes. |
| 5 | Baking and substitutions. | ‘Bake-off’ – competition to make the best chocolate cake or cookies |
|  | Energy density and satiety mechanisms, and why no calorie counting. Credit: Jeff Novick | How to overhaul a kitchen to plant based. Credit: Rip Esselstyn. |
| 6 | Diabetes: research, aetiology, role of insulin. Credit: Dr Barnard | Oil free breads, fruit bread, fruit rolls |
|  | Sleep hygiene. | Shepard’s Pie, Pavlova and fruitcakes. |
| 7 | Latest results feedback. Reflective session. | Sweet snacks from dried fruits. |
|  | Potluck – shared meal. | |
| 8 | Quiz night at local restaurant. | WFPB platters from restaurant. |
|  | Participant choice for session – exercise. Discussed common barriers to exercising. | Physiotherapist demonstrated simple stretches. Credit: Phil Watson. |
| 9 | Group discussion, reflection on successes. | Burgers and fries taught by the group mentor. |
|  | Restaurant for ‘Hot Pot’; vegetables, rice and sauce. | |
| 10 | Impact of diet on the environment. | Quiz on ‘where do you get your nutrients?’ |
|  | Latest group results. | Beetroot salad and stuffed capsicum. |
| 11 | Heart disease. Credit: Dr Esselstyn, Dr Ornish | Blueberry muffins and cinnamon scrolls. |
|  | What is mindfulness? Importance of emotional health. | Salad in a jar. |
| 12 | The plant based lifestyle and children. | Tortillas from masa flour, salsa, mexican beans. |
|  | Graduation ceremony and Potluck. Certificates for all participants. Most sessions were filmed for documentation purposes and participants received montage of video from throughout the course of the study. | |
| Post | Individual consultations repeated at week 12. Participants requested ongoing meetings after week 12, so at months 4 and 5 we had potluck dinners. At month 7 we held a feedback sessions, presented provisional results and an 18-minute film montage of the programme, and gave the participants DVD copies | |
