# Supplementary material for: The BROAD study: A randomised controlled trial using a whole food plant-based diet in the community for obesity, ischaemic heart disease or diabetes
Source: Nutr Diabetes. 2017 Mar 20;7(3):e256–. doi: 10.1038/nutd.2017.3 (PMC5380896; doi:10.1038/nutd.2017.3)
Supplement: Supplementary Table 5 [file nutd20173x8.docx]

| **Supp. Table 5.** Measurement points | | | | | |
| --- | --- | --- | --- | --- | --- |
| **Time (month)** | **0 (Enrolment)** | **0 (Baseline)** | **1 and 2** | **3 and 6** | **12** |
| Benefits and downsides | Both |  |  |  |  |
| Readiness for change | Both |  |  |  |  |
| Nutritional self-efficacy | Both |  |  | Both | Intervention |
| General self-efficacy | Both |  |  | Both | Intervention |
| Self-esteem | Both |  |  | Both | Intervention |
| Physical measurements^a^ |  | Both | Intervention | Both | Intervention |
| Blood tests^b^ |  | Both | Intervention | Both | Intervention |
| 3-day food recall |  | Both | Intervention | Both | Intervention |
| 3-day exercise recall |  | Both | Intervention | Both | Intervention |
| Food enjoyment |  | Both |  | Both | Intervention |
| Estimated food cost |  | Both |  | Both | Intervention |
| SF 36v2 |  | Both |  | Both | Intervention |
| Medications |  | Both |  | Both | Intervention |
| Photos^c^ |  | Intervention |  | Intervention |  |
| BFI 44 |  | Both |  |  |  |
| List other changes to lifestyle |  |  |  | Both | Intervention |

^a^Physical measurements were: Height *(cm)*, Weight *(kg)*, BMI *(kg/m^2^)*, Waist circumference *(cm)* and BP *(mm Hg)*. ^b^Blood tests varied; measured at all timepoints: Cholesterol total *(mmol/L)*, Triglycerides, LDL, HDL, Total: HDL *(ratio provided by laboratory)*, HbA_1c_ *(mmol/mol)*, Creatinine *(μmol/L)*, estimated glomerular filtration rates (eGFR) *(mL/min/1·73m^2^)*, Sodium and Potassium *(mmol/L)*. Measured at baseline, month 3, 6 and 12 were: full blood count, Vitamin B12 *(pmol/L)* and uric acid *(mmol/L).* Measured at baseline only (retested if abnormal) were: Tyroid stimulating hormone (TSH) *(mIU/L)* and Free T3 *(pmol/L)*, liver function tests and urea *(mmol/L)*. ^c^Photos of face, body front, and body left profile.
